# Donor Simvastatin Treatment in Heart Transplantation: 5-y Results of SIMVA Trial

**DOI:** 10.1097/TP.0000000000005491

**Published:** 2025-07-22

**Authors:** Emil Holmström, Simo O. Syrjälä, Kishor Dhaygude, Rainer Krebs, Ilkka Helanterä, Jyri Lommi, Antti I. Nykänen, Karl B. Lemström

**Affiliations:** 1 Transplantation Laboratory, Faculty of Medicine, University of Helsinki, Helsinki, Finland.; 2 Department of Cardiothoracic Surgery, Heart and Lung Center, Helsinki University Hospital, Helsinki, Finland.; 3 Department of Transplantation and Liver Surgery, Helsinki University Hospital, Helsinki, Finland.; 4 Department of Cardiology, Heart and Lung Center, Helsinki University Hospital, Helsinki, Finland.

Ischemia–reperfusion injury remains an unsolved problem in solid organ transplantation. In several preclinical studies across different organ transplants, statin treatment has been shown to mitigate ischemia–reperfusion injury.^1^ Moreover, postoperative recipient statin treatment reduces the development of cardiac allograft vasculopathy (CAV), making it a part of a routine postoperative medication protocol.^2^ In 2019, we showed in a randomized clinical trial that a single dose of simvastatin given to the donor immediately after the declaration of brain death reduced heart transplant ischemia–reperfusion injury, as measured by postoperative plasma troponin release.^3^ Furthermore, we showed that donor simvastatin administration also reduced the need for early intravenous rejection treatments. Here, we report the 5-y results of our study.

Between 2010 and 2016, we randomized 84 heart transplant donors to receive an 80-mg dose of simvastatin through nasogastric tube at the time declaration of brain death, or to control group. All heart transplant recipients received simvastatin from the first postoperative day onward. Furthermore, noncardiac organs that were retrieved from these donors and transplanted at our center were enrolled for follow-up (27 lungs, 106 kidneys, 43 livers, and 15 pancreases).

The baseline characteristics of both heart transplant donors and recipients were comparable. Mean donor age was 45 and 44 y in control and donor simvastatin groups, respectively, and 31% and 17% were female, respectively. Recipient mean age was 56 and 52 y in the control and donor simvastatin groups, respectively, with 31% and 21% being female, respectively. There were no differences in donor morbidity, but the recipients in the simvastatin group had better preoperative kidney function (estimated glomerular filtration rate 51 versus 59 mL/min/1.73 m^2^; *P* = 0.024), and perhaps because of this, lower preoperative N-terminal pro-B-type natiuretic peptide levels (3900 versus 2300 ng/L; *P* = 0.001). There were no differences in ischemia time or postoperative immunosuppressive medication between groups.

**FIGURE 1.**
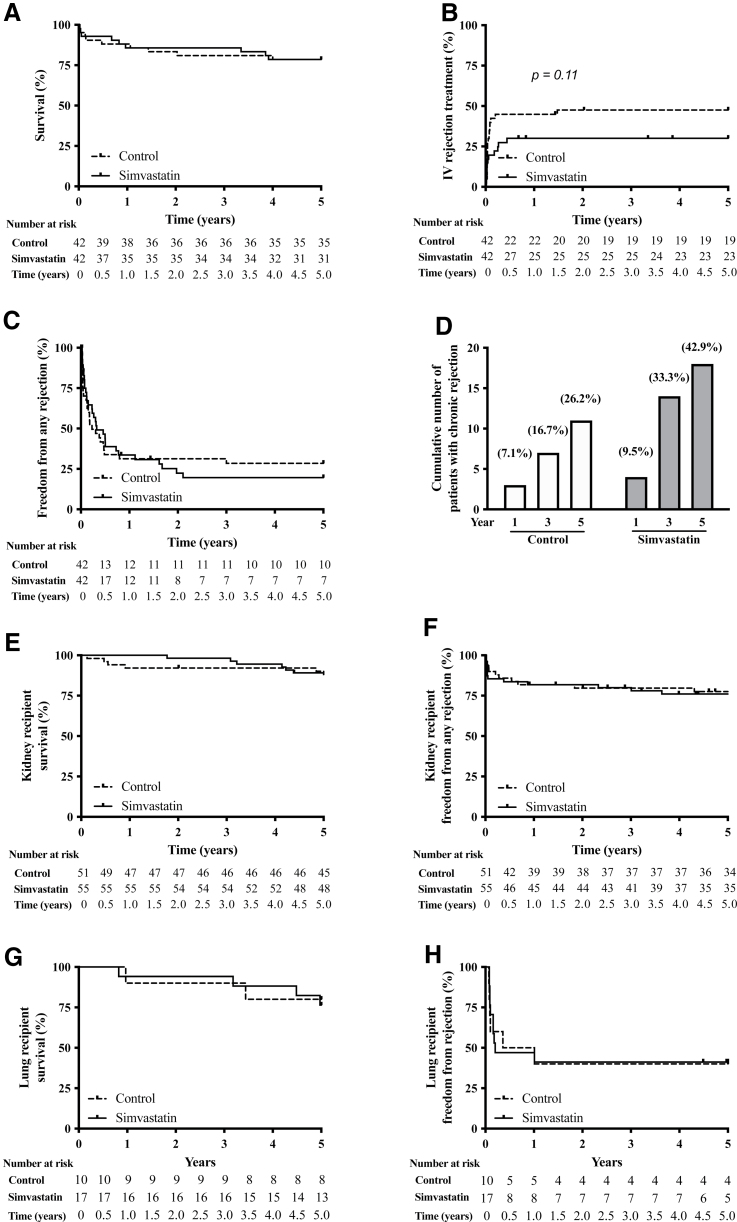
Five-year follow-up results of donor simvastatin trial. A, Overall survival in HTx recipients. B, Need of intravenous rejection treatments in HTx recipients. C, Freedom from acute rejection of any grade after HTx. D, Cumulative incidence of cardiac allograft vasculopathy at 1, 3, and 5 y in HTx recipients. E, Overall survival in KTx recipients. F, Freedom from acute rejection of any grade in KTx recipients. G, Overall survival in LuTx recipients. H, Freedom from acute rejection of any grade in LuTx recipients. HTx, heart transplantation; KTx, kidney transplantation; LuTx, lung transplantation.

The overall 5-y survival was 79% (66 of 84, with 9 patients having expired in both groups (Figure 1A). Of the 18 patients that expired during the follow-up, 7 patients died of a graft-related cause: 2 patients in both groups because of severe primary graft dysfunction, and 1 patient in the control group and 2 patients in the donor simvastatin group due to CAV. Twelve recipients developed a malignancy during the 5-y follow-up (6 recipients in both groups).

As reported previously, the control group had a higher incidence of early acute rejection episodes with hemodynamic compromise and required more intravenous rejection treatments in the first 30 d posttransplant. After the early postoperative period, however, there were no differences in the incidence of acute rejection episodes or rejection treatments between groups (Figure 1B and C). The overall incidence of CAV at 5-y follow-up was 31% (9 and 17 for control and donor simvastatin groups, respectively; *P* = 0.11; Figure 1D). Furthermore, 3 patients who expired during the follow-up period were diagnosed with chronic rejection at autopsy (2 in the control group and 1 in the donor simvastatin group).

There were no significant differences in 5-y outcomes of kidney or lung transplant recipients as measured by mortality or incidence of rejection (Figure 1E and F for kidney transplants, and Figure 1G and H for lung transplants). Liver transplant recipient 5-y outcomes have been published previously with no significant differences seen between groups.^4^

As the sample size was modest and the treatment targets the immediate perioperative phase, it is not surprising that we did not see clear beneficial long-term effects with donor simvastatin treatment. However, together with our original findings, several conclusions can be made: first, our study highlights that there is a window of opportunity between the declaration of donor brain death and organ procurement where one can intervene in the deleterious processes occurring in the donor. Second, our 5-y follow-up results suggest that single-dose donor simvastatin treatment is safe and can be implemented in routine protocol, even with multiorgan donors. Importantly, we did not notice any adverse effects from donor statin treatment in noncardiac transplant recipients.

Postoperative statin therapy is well-established in heart transplant recipients, because it has been shown to reduce the incidence of CAV.^2^ In our protocol, all heart transplant recipients received simvastatin postoperatively at a dose of 10–20 mg daily. Simvastatin was generally well-tolerated; it was switched to an alternative statin in only 11 patients (6 in the control group and 5 in the donor statin treatment group), with muscle aches being the most commonly reported side effect. However, simvastatin is primarily metabolized via the CYP3A4 pathway and may interact with other medications, particularly the calcineurin inhibitors tacrolimus and cyclosporine. Therefore, it may be prudent to consider pravastatin or rosuvastatin for postoperative use, because these agents undergo minimal or no metabolism via the cytochrome P450 system.

In noncardiac transplant recipients, all lung recipients received postoperative statin treatment, whereas statin treatment after kidney and liver transplantation was more heterogenous and depended on patient cholesterol levels. In lung transplant recipients, we did not notice any benefits from donor statin treatment; however, it has been reported that recipient preoperative statin treatment may decrease the severity of lung transplant primary graft dysfunction.^5^

In heart transplants, donor simvastatin treatment alleviated ischemia–reperfusion injury, with no significant impact on the immunological fate of the transplant in long-term follow-up, suggesting that the immunological benefits of donor simvastatin treatment are limited to the early postoperative period. Of note, however, is that statins are known to stabilize endothelial integrity and improve mitochondrial, endothelial, and myocyte function. It is possible that the reduction in the incidence of early acute rejection episodes with hemodynamic compromise may have been because of these stabilizing effects.^6^ Moreover, statins have pleiotropic immunomodulatory effects on T cell–mediated processes. Statins can inhibit major histocompatibility class II expression on antigen presenting cells, they may impair T-cell proliferation by inhibiting Ras and ρ pathways, and they can steer the immune response from Th1 phenotype towards Th2-biased phenotype, thus dampening the alloimmune response.^7^

Interestingly, a large prospective randomized multicenter study (the SIGNET study; National Institute for Health Research Health Technology Assessment Programme reference NIHR131124) is currently underway.^8^ This study used a similar donor simvastatin treatment protocol as we described here, and was powered to reveal possible long-term effects of donor simvastatin treatment.
